# Effects of a Fall Prevention Program on Fall Risks, Fear of Falling, and Depression in Older Active Adults

**DOI:** 10.1093/geroni/igaa057.755

**Published:** 2020-12-16

**Authors:** James Karnes, Victoria Bates, Ashley Chafin, Sarah Fisher, Kelcie Gilmore, Kelsey Pack

**Affiliations:** georgia southern university, savannah, Georgia, United States

## Abstract

Falls are the leading cause of injury in older adults; one in four older adults fall each year. The Otago Exercise Program (OEP) is an evidence-based fall prevention program that has been shown to reduce fall risk factors. However, exercise dosage is not known. The purpose of this study was to investigate the effectiveness of different frequencies of an OEP-based program on fall risk factors, fear of falling, and depression in older adults. Of 62 subjects initially recruited at community centers, 28 subjects met inclusion criteria and were assigned to control (Con), once-weekly (Grp 1), or twice-weekly (Grp 2) intervention groups based on subject attendance. Intervention consisted of a 12-week OEP-based program. Pre-intervention dependent variables included: 4-Stage Balance Test, 30-Second Chair Stand, Timed Up and Go (TUG), Geriatric Depression Scale (GDS), and Modified Fall Efficacy Scale (MFES). After 12 weeks, post-intervention testing assessed changes in these variables. Preliminary analysis of data using mixed design ANOVA (p.05) indicated significant changes between and within all groups for TUG and 30-Second Chair Stand. Results also suggested all groups improved on all variables with a direct relationship to exercise frequency. Furthermore, Grp2 improved more than Grp1 and Con in the 4-Stage Balance Test. These findings suggest an OEP-based falls prevention program performed 2x/week is more beneficial in decreasing fall risks and increasing lower extremity muscle strength than 1x/week. Moreover, results imply exercise frequency may be important in improving the magnitude of select falls risks variables.

